# Growth of Lactic Acid Bacteria on Gold—Influence of Surface Roughness and Chemical Composition

**DOI:** 10.3390/nano10122499

**Published:** 2020-12-13

**Authors:** Joanna Grudzień, Magdalena Jarosz, Kamil Kamiński, Mirosława Kobasa, Karol Wolski, Marcin Kozieł, Marcin Pisarek, Grzegorz D. Sulka

**Affiliations:** 1 Faculty of Chemistry, Jagiellonian University, Gronostajowa 2, 30-387 Krakow, Poland; grudzien@chemia.uj.edu.pl (J.G.); jarosz@chemia.uj.edu.pl (M.J.); mirka14.kobasa@student.uj.edu.pl (M.K.); wolski@chemia.uj.edu.pl (K.W.); marcin.koziel@uj.edu.pl (M.K.); sulka@chemia.uj.edu.pl (G.D.S.); 2 Institute of Physical Chemistry, Polish Academy of Sciences, Kasprzaka 44/52, 01-224 Warsaw, Poland; mpisarek@ichf.edu.pl

**Keywords:** *Lactobacillus* spp., nanotopography, gold substrate, polymer cation derivatives

## Abstract

The main focus of this work was to establish a correlation between surface topography and chemistry and surface colonization by lactic acid bacteria. For this reason, we chose gold substrates with different surface architectures (i.e., smooth and nanorough) that were characterized by atomic force microscopy (AFM), electron scanning microscopy (SEM), and X-ray diffractometry (XRD). Moreover, to enhance biocompatibility, we modified gold substrates with polymeric monolayers, namely cationic dextran derivatives with different molar masses. The presence of those layers was confirmed by AFM, infrared spectroscopy (IR), and X-ray photoelectron spectroscopy (XPS). In order to determine the adhesion abilities of non-modified and modified gold surfaces, we tested three lactic acid bacteria (LAB) strains (i.e., *Lactobacillus rhamnosus* GG, *Lactobacillus acidophilus,* and *Lactobacillus plantarum* 299v). We have shown that surface roughness influences the surface colonization of bacteria, and the most significant impact on the growth was observed for the *Lactobacillus rhamnosus* GG strain. What is more, covering the gold surface with a molecular polymeric film by using the layer-by-layer (LbL) method allows additional changes in the bacterial growth, independently on the used strain. The well-being of the bacteria cells on tested surfaces was confirmed by using selective staining and fluorescence microscopy. Finally, we have determined the bacterial metabolic activity by measuring the amount of produced lactic acid regarding the growth conditions. The obtained results proved that the adhesion of bacteria to the metallic surface depends on the chemistry and topography of the surface, as well as the specific bacteria strain.

## 1. Introduction

Since antiquity, some metals were considered suitable for producing self-sanitize items thanks to the oligodynamic effect [1]. It applies to situations in which metals, especially heavy metals present in a small amount, have biological activity, usually antibacterial. Nowadays, it is known that this bacteriostatic effect is induced by the surface properties of precious metals and the large specific surface area of nanostructures [2]. When it comes to the utility of metallic items and its biocidal features, the propagation of microorganisms on its surface is almost impossible, mainly due to the contact killing [3,4]. One of the most well-known metals with antibacterial properties is gold, either in the form of ions or nanoparticles [5]. Although bacteria adhesion to metallic surfaces is generally an unwanted phenomenon because of the vast demand for antibacterial materials [6,7,8,9], for some applications, e.g., in bioelectrochemical systems (BESs), the use of metal-based electrodes along with microorganisms grown on their surfaces is crucial [10].

It is known that the adhesion of microorganisms to the substrates is a complex process that depends on many biological and physicochemical factors. Among them are material surface properties such as morphology, hydrophobicity, and charge. Considering the influence of the surface morphology on the process of adhesion and growth of bacteria, the surface roughness effect gains increasing attention by researchers. A large number of studies revealed that a rough surface facilitates the adhesion of microorganisms compared to smooth materials [11,12,13,14]. One explanation of this phenomenon is that rough substrates have a large surface area, which makes it more accessible than the flat one. What is more, it was found that the surface irregularities could screen bacteria from shear forces [13]. On the other hand, Wu et al. [15] revealed that the biofilm formation was hampered by material roughness at the nano level. This observation was explained by deformation and elongation of the cell membrane on the surface unevenness. Therefore, the influence of surface topography on bacteria colonization is a complex problem and should be considered a critical factor in designing surfaces for specific purposes.

As was emphasized above, the process of bacterial adhesion is complex and cannot be generalized for all bacterial strains. The nature of bacteria and its specific binding mechanism to the material surface (bacteria-substrate interaction) has a critical role [13]. That is why, despite the antimicrobial properties of precious metals mentioned earlier, some bacteria species were found to live in the presence of gold. For instance, *Geobacter sulfurreducens* formed a biofilm on the surface of the gold electrode after one month of operation in microbial fuel cells [16]. Another example is *Cupriavidus metallidurans* strain, which can use gold compounds derived from the environment to synthesize golden nuggets [17]. What is more, our previous study revealed that the material based on an electrochemically produced gold layer covered with a cationic polysaccharide dramatically improved the adhesion of *Lactobacillus rhamnosus* GG bacteria and promoted its growth [18], which may be considered as one more exception from the generally accepted assumption concerning gold materials. These Gram-positive bacteria can be found in the human gastrointestinal tract (GIT) and are commonly used to produce probiotics [19] because of their beneficial health effects. For that reason, the majority of studies refer to the process of adhesion of *L. rhamnosus* GG solely to the gastrointestinal epithelial cells [20], and so far, there is still a lack of detailed studies concerning colonization of metallic materials by this strain.

It is worth mentioning that the bacteria’s ability to adhere and form biofilm can differ within a particular bacteria strain and within bacteria phenotype [21,22]. It was found that the structure of a cell wall of Lactobacillus genera may vary, which strongly affects cell–host interactions [21]. What is more, Polak-Berecka et al. [22] investigated the dependence between the cell envelope components of two *Lactobacillus rhamnosus* phenotypes (E/N and PEN) and the process of adhesion to the human cell line. They concluded that variation at the degree of adhesion was associated with the composition and structure of bacterial cells. That is why the second issue addressed in this work is demonstrating whether the influence of the surface roughness on the adhesion process can be generalized to other strains of lactic acid bacteria from *Lactobacillus* species (LAB). Therefore, *Lactobacillus acidophilus* and *Lactobacillus plantarum* 299v were also selected to conduct the presented studies. Similarly to *L. rhamnosus* GG, the chosen bacterial strains are commonly used as probiotics and are inhabitants of the human GIT [23]. These selected strains like most of the lactic acid bacteria prefer existences in suspension form, except for a few specific strains (*Lactobacillus kunkeei* [24], *Lactobacillus sakei* [25]) but in recent years, there have been reports in the literature that they can be forced to form biofilms or biofilm-like structures [26,27].

Various kinds of modifications may alter the ability of the bacteria attachment to a material surface. In the case when bacterial adhesion is the unwanted process, covering the surface with a layer of poly(ethylene glycol) (PEG) and its derivatives or with self-assembled monolayers (SAMs) of, e.g., alkanethiols is typically carried out [28,29,30]. On the other hand, there are known systems that require microorganisms on the substrate surface. Our previous research demonstrated that modification of the negatively charged gold layer with a cationic polysaccharide might affect the attachment and proliferation of *Lactobacillus rhamnosus* GG bacteria [18]. A similar strategy was used for new material, but currently, the effect of the molecular weight (6, 40, and 100 kDa) of cationic dextrans on surface interactions with the lactic acid bacteria strains will also be studied. It is known that such polycations have the ability to model biological activity and human cell growth [31,32], which may be related to the interaction with a negatively charged biological membrane or negatively charged proteins and polypeptides.

In this work, we describe in detail the growth phenomenon of commonly occurring lactic acid bacteria strains on the gold surface. This research is aimed at investigating (i) the ability of lactic acid bacteria (*L. rhamnosus* GG, *L. plantarum* 299V, and *L. acidophilus*) to adhere to and proliferate on the gold surfaces; and (ii) the influence of the surface nanoroughness of the gold material and surface modification (coverage with polycation nanolayer) on the adhesion and proliferation processes. To the best of our knowledge, such a detailed characterization of the colonization of different LAB strains on gold surfaces is shown for the very first time and will help gain insight into the processes governing bacteria growth on metallic surfaces. This, in turn, will lead to more efficient preparation of surfaces for various applications that utilize bacteria.

## 2. Materials and Methods

### 2.1. Materials

Gold (200 nm)-covered glass discs (batch H103-196) were acquired from Ssens bv (Enschede, The Netherlands). Copper foil (99.9%, 0.5 mm thick) was purchased from Krysmet (Brzezie, Poland). Acetone, sulfuric acid, and phosphoric acid were obtained from Chempur (Piekary Śląskie, Poland), while ethanol, methanol, sodium citrate, citric acid, and phosphoric acid from POCh (Gliwice, Poland). MRS Broth, phosphate-buffered saline tablets (PBS), dextrans (6, 40, and 100 kDa), glycidyltrimethylammonium chloride (technical, ≥90%), hexamethyldisilazane, crystal violet, and Lactate Assay Kit were purchased from Sigma-Aldrich (Poznań, Poland). Glutaraldehyde (25%) was obtained from Acros Organics (Belgium). The following strains of lactic acid bacteria were used in this work: *Lactobacillus rhamnosus* GG (Dicoflor^®^60, Bayer, Leverkusen, Germany), *Lactobacillus acidophilus* (Swanson, Fargo, ND, USA), *Lactobacillus plantarum* 299v (Sanprobi IBS, Sanum, Hoya, Germany). A commercially available gold solution Auruna^®^556 was acquired from Umicore (Région de Bruxelles-Capitale, Belgium). Fluorescent imaging of microorganisms was performed by using the LIVE/DEAD™ Baclight™ Bacterial Viability Kit (Invitrogen, Thermo Fisher Scientific, Waltham, MA, USA).

### 2.2. Synthesis of Gold Layers on a Metal Substrate

Copper samples covered with a gold layer (Au(E)) were synthesized by modifying the previously reported procedure [18]. Briefly, a copper foil was cut into coupons (2 cm × 1 cm) and degreased in acetone and ethanol. Samples were polished, first electrochemically in a mixture of phosphoric acid and water (5:2 *v*/*v*) at the current density of 0.3 A·cm^−2^ for 2 min, and then chemically in 0.5 M sulfuric acid for 1 min, rinsed with distilled water and ethanol, and dried in air. Next, as-prepared electrodes were covered with a thin gold layer by sputter deposition (Quorum Q150T S sputter-coater, Quorum, Sussex, UK). In contrast to the previous procedure [18], a different commercially available gold solution (Auruna^®^556, 12 g·L^−1^ Au) was used to thicken the gold layer. An electrochemical deposition was carried out for 600 s at the constant current density of 1.5 mA·cm^−2^ by using a potentiostat (SP300, Biologic, Seyssinet-Pariset, France) in a conventional three-electrode cell, where a copper/sputtered gold substrate, platinum grid, and platinum wire were used as a working, counter, and reference electrodes, respectively. The process was conducted at a temperature of 50 °C, and the evaporated solution was refilled continuously with distilled water.

### 2.3. Synthesis of Polycations

Three cationic dextran derivatives of different molecular weights were selected in this work to modify the gold surface by layer-by-layer (LbL) deposition. They were obtained using commercially available raw dextran with masses of 6, 40, and 100 kDa, respectively, and cationization were carried out using glycidyltrimethylammonium chloride (GTMAC). Details of the synthesis were described in our earlier work [33]. Briefly, for all dextrans, 2 g of polymer was dissolved in 100 mL of water, and 12 mL of GTMAC and 400 mg of sodium hydroxide were added to the solution. The synthesis was carried out at 60 °C for 4 h. After completion, the product was purified by dialysis into water and lyophilization.

Zeta potential measurements were performed by using the Zetasizer Nano ZS (Malvern Panalytical, Malvern, UK). The concentration of tested polymer solutions was 5 g·L^−1^. The used solvents were deionized water (conductivity 0.5 µS) and isotonic PBS with pH = 7.4.

### 2.4. Surface Modification of Gold Substrates

The Au(E) and gold on glass (Au200, 200 nm) were subjected to further surface modifications with polycations mentioned in paragraph 2.3. by using the LbL method. After 5 min of UV (Low-pressure Hg lamps UV-C) sterilization, the obtained gold substrates were immersed in solutions containing 0.1 g·L^−1^ of each polymer in an isotonic phosphate buffer solution (PBS, Ph = 7.4) for 10 min. As control samples, unmodified Au(E) and Au200 plates were used, immersed only in a PBS solution. Subsequently, the polymer excess was removed by rinsing the sample surface several times with small PBS portions. Synthesized samples were name-coded Dex6, Dex40 and Dex100 for 6, 40 and 100 kDa respectively. 

### 2.5. Bacteria Inoculation and Cultivation

**Figure 1:**
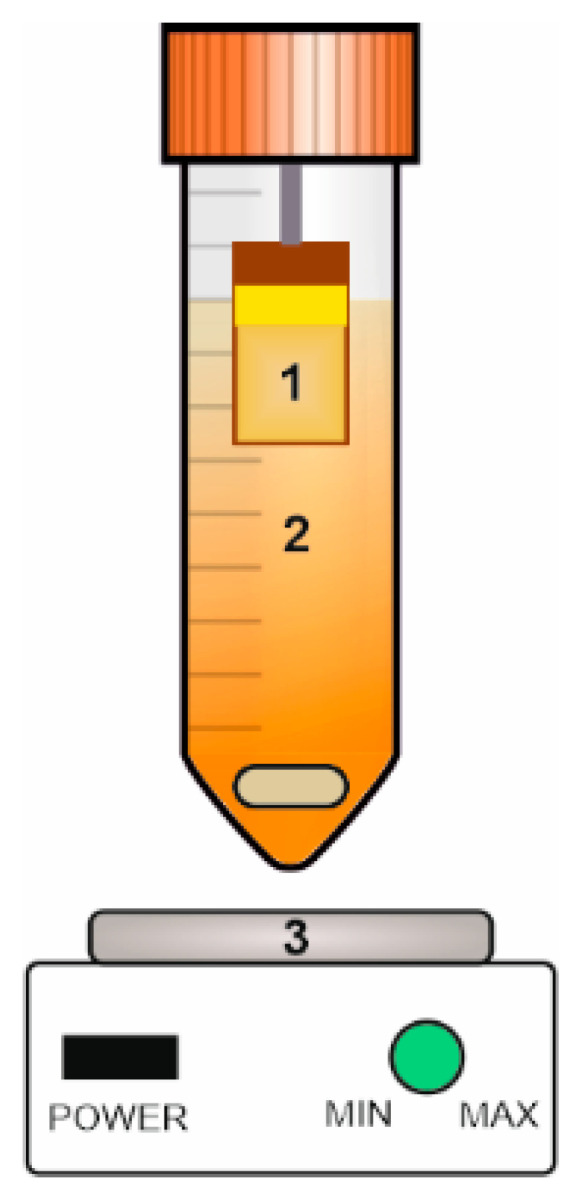
Schematic representation of the inoculation process, where: 1—colonized surface; 2—MRS Broth medium containing bacteria inoculum (OD = 1); 3—magnetic stirrer.

The culture medium used in this study, for each selected bacteria strain, was MRS Broth prepared without any modification to the commercial protocol. An inoculate was obtained by culturing each bacteria strain in the medium for 24 (for *L. rhamnosus* GG and *L. plantarum* 299v) or 48 h (for *L. acidophilus*) at 20 °C. In order to estimate the same start-point for colonization of gold surfaces, the inoculate was diluted with a fresh culture medium so that the optical density (OD) was equal to 1. The OD measurements were performed by using a UV-Vis spectrophotometer (Evolution 220, Thermo Scientific, Waltham, MA, USA) at 600 nm wavelength in a quartz cuvette with an optical path length of 1 cm. The diluted inoculate was transferred to a Falcon tube with an Au(E) or a Au200 substrate attached to its upper part (see Figure 1). In the bottom part of the vessel, a stirring bar providing homogenous dispersion of the inoculate was placed. The gold substrate was left in a diluted suspension for 30 min allowing bacteria to adhere. It is worth mentioning that the designed system ensures, by the vertical arrangement of studied materials, deposition of the microbes only due to the affinity to the surface, not due to gravity-driven sedimentation.

Then, the plate (Au(E) or Au200) was transferred to a new Falcon tube with a fresh medium, where bacteria were cultured for 48 h (unless said otherwise) without stirring. The medium was changed every 24 h by transferring the sample to the Falcon tube with a fresh medium. All procedures were performed in sterile conditions.

### 2.6. Surface Characterization

The surfaces of the tested materials (with and without the polymer layers) were characterized by using several imagining techniques. The roughness of surfaces was determined by means of atomic force microscopy (AFM). For this, a Dimension Icon AFM (Bruker, Santa Barbara, CA, USA) working in the PeakForce Taping (PFT) and QNM modes was used with standard silicon cantilevers for measurements in the air (nominal spring constant of 0.4 N·m^−1^). Moreover, in order to assess the crystallinity of the gold layers, the X-ray diffraction (XRD) measurements were performed on Au(E) samples and a commercially available gold layer on the glass as a reference sample (Panalytical X’Pert PRO MPD, λ_1_ = 1.5406 Å, λ_2_ = 1.5444 Å; λ_1_/λ_2_ = 2:1). The surface chemistry of the uncoated and coated Au(E) samples was analyzed using X-ray photoelectron spectroscopy for chemical analysis (ESCA-XPS) and infrared spectroscopy (IR). For XPS measurements, a Microlab 350 (Thermo Electron, Waltham, MA, USA) spectrometer with non-monochromatic Al Kα radiation (hν = 1486.6 eV, power 300 W, voltage 15 kV) was used for investigations. The analyzed area was 2 × 5 mm. The hemispherical analyzer was used for collecting the high-resolution (HR) XPS spectra with the following parameters: Pass energy 40 eV, energy step size 0.1 eV. The collected XPS spectra were fitted using Avantage software (version 5.9911, Thermo Fisher Scientific, Waltham, MA, USA), where a Smart function of background subtraction was used to obtain XPS signal intensity. For this purpose, an asymmetric Gaussian/Lorentzian mixed function was applied. The position of the carbon C1s peak was assumed to be at 285.0 eV and used as an internal standard to determine the binding energy of other photoelectron peaks. The IR measurements for polymer-coated samples were captured using the Nicolet iS10 FT-IR spectrometer with a grazing-angel reflectance accessory (at an incident angle 80°) under the p-polarized beam. All the spectra were baseline corrected using OMNIC software. Finally, the surface characterization of the samples without bacteria was also performed using scanning electron microscopy imaging (SEM, Hitachi S-4700) with energy-dispersive X-ray spectroscopy (EDS).

### 2.7. Characterization of Lactobacillus spp. Bacterial Network on Gold Surfaces

The viability of microorganisms on Au(E) or Au200 plates with and without different polymer coatings was assessed with the LIVE/DEAD™ Baclight™ Bacterial Viability Kit. The staining mixture was prepared according to the manual provided by the supplier. Briefly, equal volumes of SYTO 9 and propidium iodide (PI) stains were combined in an Eppendorf tube and diluted in ultrapure water (Mili Q) to the final volume of 1 mL. Afterward, two drops of the mixture were placed on the sample with bacteria (after 24 h of culturing) and left for 30 s. The sample was then placed on the slide glass and observed under a fluorescence microscope (DMi8 Leica, KAWASKA, Zalesie Górne, Poland). The green (480/500 nm) and red (490/635 nm) fluorescence were observed using FITC and RHODO filters, respectively. The obtained images were analyzed using the Leica Application Suite (LAS) X and Image J [34] software packages. For each sample, 10 separate images on each channel were taken. The measurements were made in triplicates.

SEM imaging of the samples with microorganisms was also carried out, preceded by the fixing procedure described elsewhere [35,36]. Briefly, the samples were fixed in glutaraldehyde in the PBS solution (1:7 *v*/*v*) for 24 h, rinsed twice with PBS (15 min each), and dehydrated in ethanol solution series with increasing the ethanol concentration from 50 up to 100%. Finally, the samples were rinsed with hexamethyldisilazane and dried in air. Prior to microscope observations, the samples were sputter-coated with a thin gold layer (approximately 15 nm).

Additionally, an assessment of the number of bacteria on the surface was performed using Gram’s method (Gram staining). First, after 48 h of bacteria cultivation, microorganisms were fixed according to the procedure for SEM measurements. The selected surfaces were then immersed in a crystal violet (CV) solution (0.1% in 70% ethanol and water) for 10 min. After this time, the unbound dye was rinsed with distilled water, and the surface was dried with a stream of air. Next, the bacteria-bounded dye was dissolved in 1 mL of the solution obtained by dissolving citric acid (13.22 g) and sodium citrate (10.88 g) in a mixture of 500 mL distilled water and 500 mL methanol, and the absorbance at 540 nm was measured with the Epoch™ 2 Microplate Spectrophotometer (Biotek Instruments, Inc., Winooski, VT, USA).

### 2.8. Determination of Produced Lactic Acid

The lactic acid (LA) concentration secreted by microorganisms during cultivation was determined by using the Lactate Assay Kit. All reagents and samples were at room temperature. The Lactate Enzyme Mix was diluted with 220 µL of the Lactate Assay Buffer. A calibration curve was generated by adding 0, 2, 4, 6, 8, and 10 µL of the 1 nmol·µL^−1^ Lactate standard (prepared by mixing 10 µL of the 100 nmol·µL^−1^ Lactate standard and 990 µL of the Lactate Assay Buffer) into wells of the 96-well plate and filling up to the volume of 50 µL with the Lactate Assay Buffer. The optimal amount of culture medium (200- or 400-fold dilutions with the Lactate Assay Buffer) collected after 24 h-long culture were added into wells of the 96-well plate and diluted with the Lactate Assay Buffer analogously as described above. The mixture of the Lactate Assay Buffer (46 µL), the Lactate Enzyme Mix (2 µL), and the Lactate Probe (2 µL), named Master Reaction Mix, was added to each well with standards and samples solutions. After shaking and leaving for 30 min (reaction incubation time), the colorimetric measurements of the absorbance at 570 nm were performed with the Epoch™ 2 Microplate Spectrophotometer (Biotek Instruments, Inc., Winooski, VT, USA). The measurements were done in triplicates.

## 3. Results

### 3.1. Impact of the Surface Roughness on Bacteria Adhesion

**Figure 2:**
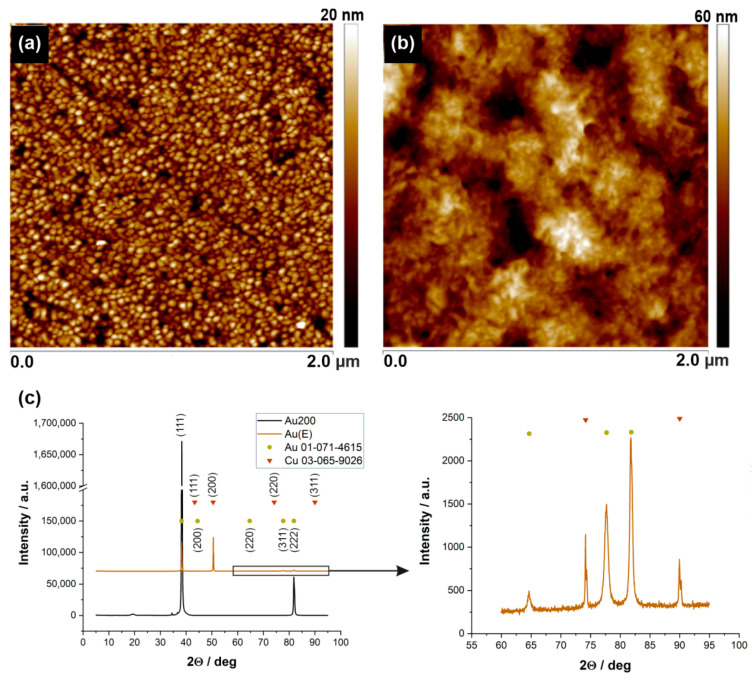
2D AFM topography images of (**a**) commercially available gold layer (200 nm) on the glass; (**b**) Au(E) obtained electrochemically. (**c**) XRD patterns of Au200 and Au(E) substrates with characteristic reflections from gold and copper.

As mentioned in the introduction section, it is well known that both surface topography and its chemistry play an important role in the adhesion process of microorganisms. For that reason, two gold substrates with different surface roughness were selected for experiments, i.e., the commercially available gold layer on glass (Au200) and the electrochemically synthesized gold layer on the copper substrate (Au(E)). Firstly, the surface morphology and crystallinity of both substrates were examined by using AFM and XRD, respectively. The obtained results are shown in Figure 2.

AFM images revealed that the differences in the surface morphology of materials are significant. The Au(E) plates have a heterogeneous topography with micro- and submicrometric scale domains (Figure 2b). On the other hand, Au200 samples can be described as smooth and with a relatively repeatable nanoscale surface morphology (Figure 2a). Moreover, the distinction between smooth and rough surfaces is also visible in the roughness parameter (R_a_), i.e., 2.4 and 7.0 nm for Au200 and Au(E), respectively.

To get an insight into the nature of the gold coverage, XRD measurements were adapted. As can be seen from Figure 2c, the Au200 samples revealed sharp reflexes of gold (at 38° and 82° for (111) and (222) planes, respectively), which indicate that gold crystals are surface-oriented with the same crystallographic plane. This may contribute to the surface smoothness visible in the AFM image. When it comes to the Au(E) sample, the diffractogram shows reflexes for both copper and gold; however, the shape of the signals from Au indicates its polycrystalline nature. Therefore, during electrodeposition, Au crystals grow in different directions at various speeds, which results in higher surface roughness, as shown in the AFM image.

**Figure 3:**
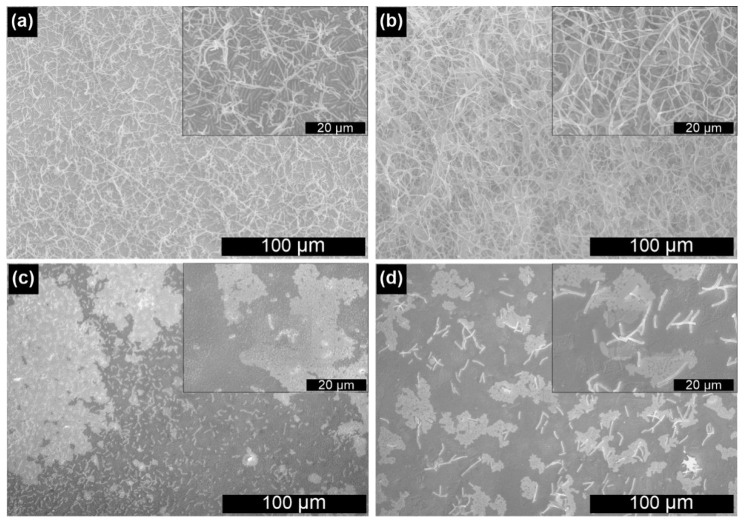
SEM microphotographs of the bacteria biofilm after 48 h cultivation: *L. rhamnosus* GG on the surface of (**a**) Au200 and (**b**) Au(E); (**c**) *L. plantarum* 299v and (**d**) *L.* acidophilus on the surface of Au(E).

The gold substrates were further tested in regard to the adhesion of bacteria cells. As mentioned earlier, three *Lactobacillus spp.* were chosen for these experiments, i.e., *Lactobacillus rhamnosus* GG, *Lactobacillus plantarum* 299v, and *Lactobacillus* acidophilus. The SEM images of examined surfaces after 48 h cultivation are shown in Figure 3.

As presented above, for both tested materials, the growth of *L. rhamnosus* GG on gold substrates is significant. After adhesion, microorganisms grew, spread, and proliferated on all available areas, forming a lace-like network, contrary to our previous work, where such a structure was observed only for modified electrodes [18]. It might be related to the different composition of the gold-rich electrolyte used for the deposition process or extended cultivation time. However, the difference between the smooth and rough Au is visible in the SEM images since the structure of the biofilm is denser on the polycrystalline surface. Also, slightly higher mortality was observed on the Au200 substrate, which will be described later. Such behavior is not typical for the growth of other bacteria strains on nanorough gold surfaces. As shown by Nguyen et al. [37], nanoscaled topography significantly reduces the adhesion of *Pseudomonas aeruginosa* (also a rod-shaped bacteria) when compared to the smooth gold substrate. Moreover, when it comes to other *Lactobacillus* strains, the surface coverage on the Au(E) substrate is significantly different (Figure 3c,d). Not only the coverage of the surface was much smaller, but also the percentage of the damaged bacteria cells increased in the order *L. rhamnosus* GG < *L. plantarum* < *L. acidophilus* (see in the next section).

**Figure 4:**
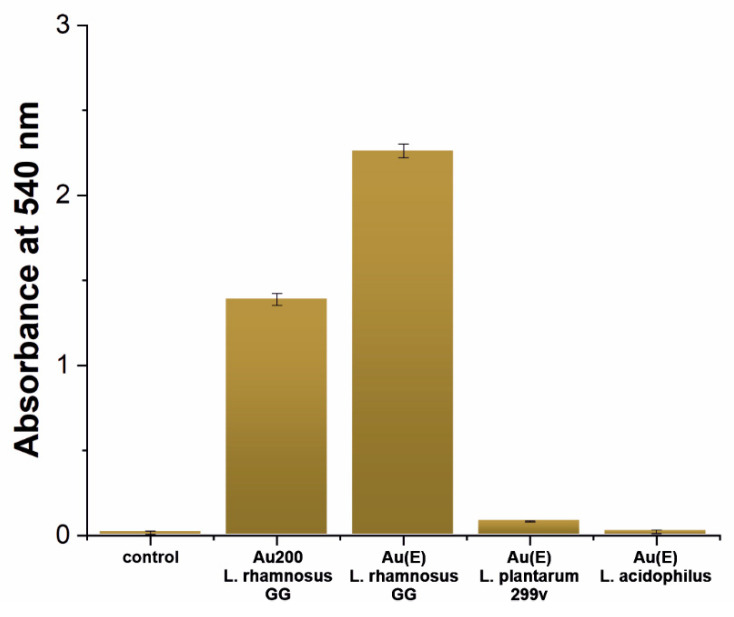
The absorbance of the crystal violet from the gold substrates after inoculation with Lactobacilli strains and corresponding photographs of the stained samples.

In order to gain more quantitative insight into the surface coverage, the Gram method was employed to determine the amount of bacteria on the surface for selected systems and confirm whether the distribution of microorganisms in the macro scale is uniform. The samples were prepared in the same way as those for SEM imaging, which enabled the direct comparison of the results. The obtained data are shown in Figure 4 and Figure S1.

The obtained results proved that in the case of the *L. rhamnosus* GG strain, an almost ideal surface coverage is obtained for the Au(E) substrate, while for Au200, the measured absorbance, and therefore, the coverage, is significantly lower (Figure 4 and Figure S1). These results confirm that in the case of *L. rhamnosus* GG, the surface nanoroughness enhances the colonization of gold. When it comes to other Lactobacilli strains, the surface coverage is almost negligible, especially for *Lactobacillus acidophilus*, where the absorbance is at the control level. Such results may be explained by the presence of the pili in the cell wall of *Lactobacillus rhamnosus* GG, which are responsible for the adhesion process [38,39]. Such adhesive proteins were not determined for the other two strains. The fact that *Lactobacillus rhamnosus* GG is not adhering using its entire surface but only with nano-objects scattered over the cell surface, allows us to conclude that this process will be critically sensitive to the specific surface of the substrate. This could explain why, for rough surfaces, we have observed more bacteria. Another aspect that could explain it is the characteristic 3D lace multi-layer structure created by bacteria on the surface. Such an arrangement provides more points of contact with the surface, which will significantly favor rougher surfaces.

Considering the results presented above, it might be stated that the colonization of the metallic surfaces by microorganisms, especially gold, depends not only on the surface properties of the material (roughness in particular), but also on the individual properties of bacteria species, including the structure of the cell wall.

### 3.2. Dextran Derivatives as Additional Surface Modifying Agents

A negatively charged gold surface may affect the adhesion of bacteria cells because their biological membranes also have a negative potential. This effect can be eliminated by using polycationic films deposited by using the layer-by-layer technique. It is a well-developed and straightforward method that allows covering a negatively charged surface with a molecular layer of the cationic polymer [40]. This leads to a change of the surface charge and, in consequence, may influence its adhesive properties. Three cationic dextran derivatives differing in a molar mass confirmed and characterized by gel permeation chromatography (GPC) method (detailed data are shown in the Supplementary Material, Figure S2) were selected for this study. In our earlier publications [31,32], we have discussed the effect of positive charge and molecular weight of polysaccharides on their biological activity and toxicity. Due to the electrostatic mechanism of nanocoating formation on the surface of gold, we imposed the synthesis conditions that the polymer charge should be fixed while the molar mass will be a variable. Such an approach is also justified by the fact that the charge will affect the toxicity, which may distort the obtained results [32]. Therefore, the zeta potential of the respective solutions was measured to confirm that we have obtained polymers with the desired charge. The results are shown in Table 1.

**Figure 5:**
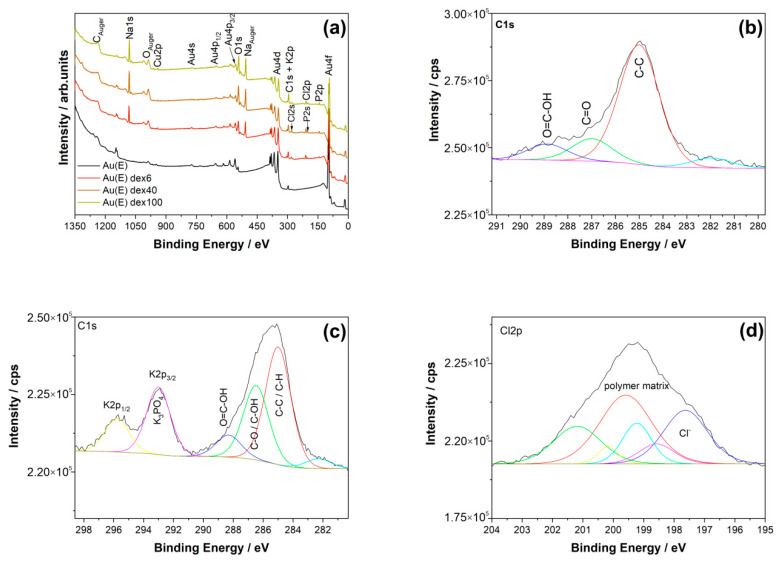
(**a**) XPS spectra for Au(E) samples, unmodified, and modified with cationic dextran derivatives. High-resolution deconvoluted XPS spectra of C 1s for (**b**) unmodified and (**c**) modified Au(E)dex40 sample, and (**d**) Cl 2p spectra for the coated sample.

The obtained zeta potential values allow us to state that the assumed goal has been achieved, and the charges of obtained polymers differ insignificantly and these differences can be regarded as negligible. The effectiveness of Au(E) substrates coverage by polymer coatings was confirmed by XPS (Figure 5), IR, and AFM (Figures S3 and S4 in the Supplementary Material) measurements.

An overview of the survey spectra for the produced samples is shown in Figure 5a. A detailed examination of the XPS spectra in relation to the unmodified samples (Figure 5b) showed that characteristic carbon C1s peaks are well visible for samples with dextran derivatives. In particular, typical C–C/C–H (285.0 eV) and C–O/C–OH (286.5 eV) bonds can be observed for these materials (Figure 5c). A peak corresponding to the caroxyl groups observed at 288.3 eV results from oxidation of carbon impurities at the surface. Moreover, the presence of a Cl 2p peak at 199.6 eV, which is a part of the polymer matrix, was found (Figure 5d). The presence of Cl in the spectra comes from the modification procedure, where glycidyltrimethylammonium chloride (GTMAC) was used in the synthesis of cationic derivatives of dextran. Although no peaks from N1s (at around 400 eV) were present, the Cl 2p spectra demonstrate that the modification was performed successfully. Also, peaks from phosphorus, sodium, and potassium were present due to the preparation procedure (see paragraphs in Section 2.2, Section 2.2, Section 2.3). Additionally, for all the prepared samples, the characteristic XPS gold (Au4f ~83.0 eV) and copper (Cu2p ~932.0 eV) peaks related to the presence of the Au coating on the Cu substrate were also visible (Figure 5a).

The presence of the dextran derivative monolayer on the Au(E) substrate was also confirmed by the IR spectra (Figure S3). Similarly to the previously reported data [18], the characteristic peaks for dextran derivatives were observed for the polymers with higher molecular mass. In particular, the O-H, C-H, and C-C stretching bands were observed at 3394 cm^−1^, 2941 cm^−1^, and 1272 cm^−1^, respectively. Moreover, the CH_3_-N bond was also visible at 1462 cm^−1^, ascribed to the C-H bending vibrations.

What is more, the AFM measurements showed that due to the small amount of the deposited polymer, the surface is slightly smothered when compared to the uncoated Au(E) substrate, but still rougher than the pristine Au200 substrate (Figure S4).

**Figure 6:**
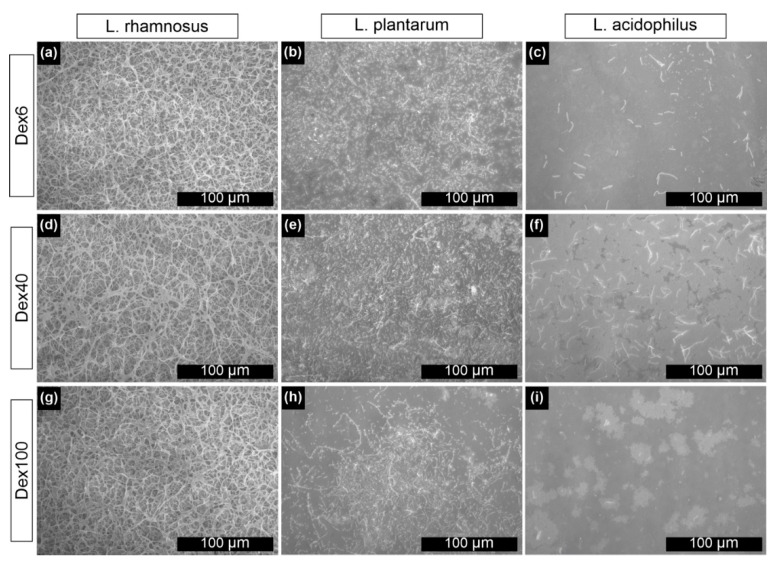
SEM microphotographs of Au(E) with a layer of (**a**–**c**) Dex6, (**d**–**f**) Dex40 or (**g**–**i**) Dex100 and (**a**,**d**,**g**) *L. rhamnosus* GG, (**b**,**e**,**h**) *L. plantarum* 299v and (**c**,**f**,**i**) *L. acidophilus* biofilms after 48 h inoculation.

Furthermore, the biocompatibility of the modified gold surfaces was assessed using the same *Lactobacilli* strains as for the non-modified samples. The SEM microphotographs of Au(E) substrates covered with polymer layers after 48 h of cultivation are shown in Figure 6.

Based on the SEM microphotographs, it may be stated that for *Lactobacillus rhamnosus* GG strain cultured on all types of modified Au(E) samples (Figure 6a,d,g), no significant differences were observed when compared to the non-modified ones (see Figure 3b). Bacteria were spread on the whole surface, and a well-developed bacterial network was present. No negative impact of the polymeric layer on the bacteria was observed, which is in good agreement with our previous work [18]. In the case of *Lactobacillus plantarum* 299v, a visible improvement in the surface coverage was observed for all types of modifiers (Figure 6b,e,h). On the other hand, for *Lactobacillus acidophilus,* a decrease (Figure 6c,i) or no change (in the case of Dex40) (Figure 6f) in the surface colonization was observed.

The presented results showed that by modifying the metallic surface with even a monolayer of biocompatible substance, an improvement in the bacteria adhesion is evident. However, it should be underlined that this dependency is not unambiguous for all strains, so the reasons behind such different behaviors should be further examined.

For all tested surfaces variants for the *Lactobacillus rhamnosus GG* strain, an additional observation is that we are not dealing with a system as stable as a classic biofilm, and bacteria can be detached in both cases (Au(E) and Au 200) with the help of a cell scraper. The surfaces covered with bacteria can be removed and re-immersed in the liquid without any detachment, but other operations, such as heavy rinsing, affect the stability of both systems in a similarly negative way.

This poor grip to the surface is due to the fact that only a few bacteria growing in the lowest layers of the 3D structure are bound to the substrate, and most of the cells are only bound to each other. It can be considered as a disadvantage, but this strain does not occur usually in nature in the form of a biofilm and cannot exist as a colony of bacteria; therefore, this may be the only form in which they can appear in a more massive cluster. Additionally, thanks to such a 3D lace multi-layer structure, bacteria have large access to nutrients, and even those individuals furthest from the surface can live without limitations.

### 3.3. The Influence of the Surface on the Welfare of Microorganisms

**Figure 7:**
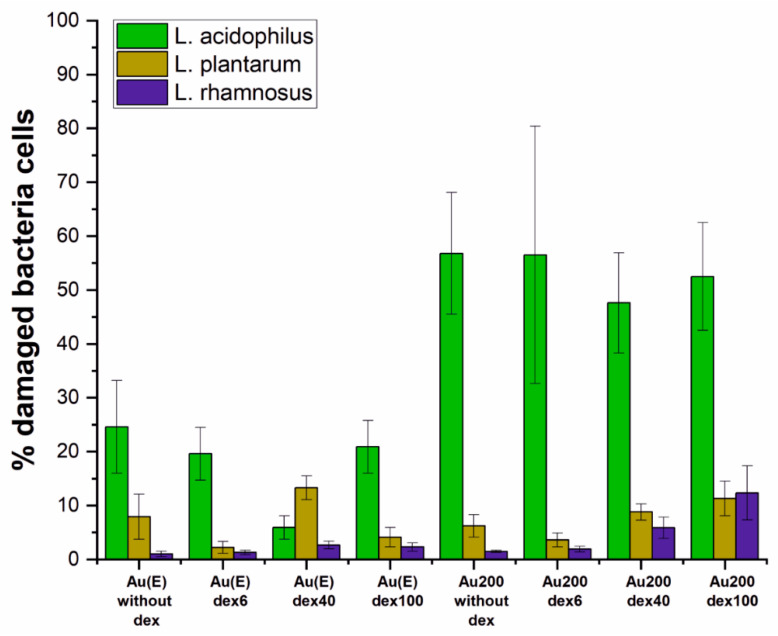
Contribution of damaged bacteria in the population that colonized the surfaces after 24 h of culture based on the live/dead staining for the fluorescent microscopy.

Based on the SEM measurements, one can only assess the degree of coverage of the surfaces by microorganisms. To assess their welfare, experiments on the non-fixed bacteria cells were conducted. All of the examined surfaces were used for fluorescent live/dead staining to establish the percentage of damaged bacteria cells on each surface and for all bacteria strains (Figure 7).

**Figure 8:**
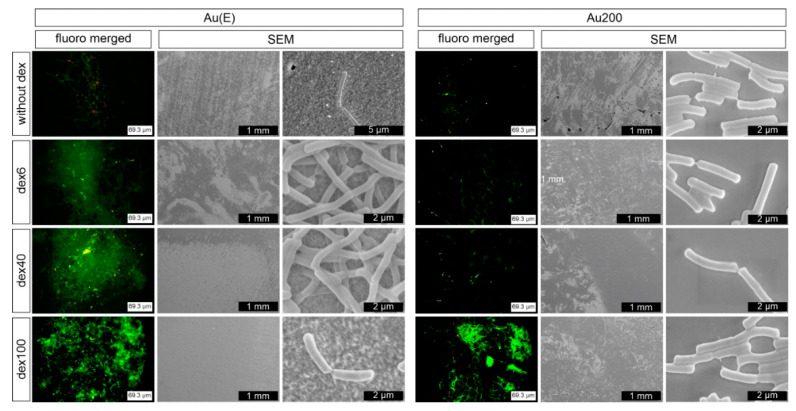
Fluorescence and SEM microphotographs of *Lactobacillus rhamnosus* GG after 24 h of incubation at Au(E) and Au200 substrates with and without polymer modifications.

As already described for the uncovered gold layers, the number of damaged bacteria cells varied between strains; however, the tendency for smooth and rough gold was the same, i.e., the highest percentage of damaged bacteria cells was observed for *Lactobacillus acidophilus*. When the samples were modified with cationic dextran derivatives, the situation seems to be more complex. For *Lactobacillus rhamnosus GG*, cells’ viability was slightly improved or remained at a similar level for the nanorough Au(E) samples. However, it should be pointed out that significantly higher surface coverage was observed on the dextran-modified than unmodified samples, which can be seen on microphotographs from the fluorescent and scanning electron microscopes (Figure 8). On the other hand, when the Au200 substrate was modified, an increase in the percentage of damaged bacteria cells was observed. Interestingly, similar tendencies were also observed for the other two tested strains on both gold surfaces, which was also confirmed microscopically (see Figures S5 and S6 in the Supplementary Materials). Such results may indicate that the enhancement of the adherence and proliferation of the bacteria cells due to the presence of dextran derivatives may also be species dependent.

Another indicator for the well-being of the examined bacteria on the tested substrates is the amount of produced lactic acid, a metabolite of all three tested bacteria strains. It should be mentioned that in the experimental setup, bacteria live both on the gold surface and in the surrounding medium (due to the detachment of some of the bacteria during cultivation). Therefore, their metabolic products are present in the medium used for the determination of LA. Obviously, such an approach is not ideal, but it gave us an insight into the biological activity of the entire system. The results are shown in Table 2, and the calibration curve is shown in Figure S7.

As shown, for *L. acidophilus* strain, the measured concentrations are at the limit of detection, so it is hard to clearly confirm the bacterial activity/presence on the surface. On the other hand, *L. rhamnosus* and *L. plantarum* generated a significant amount of LA that further proves that live bacteria are present and maintain their metabolic activity. What is more, the results obtained for LA production also confirmed that the proposed modifications are the most beneficial for *Lactobacillus rhamnosus* GG, whereas, for other strains, the results are inconclusive.

Nonetheless, this demonstrates that the tested substrates are not toxic towards the Lactobacilli strains; however, when designing a biosurface for specific applications, many factors that could affect its performance should be assessed.

## 4. Conclusions

Interaction between metallic surfaces and microorganisms may be a critical issue in many applications, e.g., in bioelectrochemical systems. That is why it is crucial to determine the conditions under which the colonization of bacteria on the desired surface is optimal. Our study showed that the colonization of the gold surface depends on its topography; however, contrary to most studies, it is better when the surface roughness is increased. Moreover, by changing the surface chemistry, i.e., covering gold with a monolayer of cationic dextran derivatives, a slight improvement in the bacteria adhesion and proliferation may be observed. Interestingly, the obtained results are also species-related, which may be ascribed to their cell wall structures, especially the lack or presence of the adhesive pili fibers. However, further studies, preferably on the molecular and biochemical levels, should be applied to assess the reasons behind such behavior.

## Figures and Tables

**Table 1:** Zeta potential of tested polymers depending on the used solvents.

Solution	Zeta Potential/mV		
	Dex6	Dex40	Dex100
Water solution	24.00 ± 6.35	31.70 ± 1.45	27.40 ± 3.25
PBS solution	−4.85 ± 0.84	−4.93 ± 0.33	−6.99 ± 0.45

**Table 2:** The average concentration of lactic acid (nmol·L^−1^) depending on the substrate and polymer after 24 h of cultivation of lactic acid bacteria strains.

Sample Name	*L. rhamnosus*	*L. plantarum*	*L. acidophilus*
	Concentration nmol·µL^−1^ ± 5.7 nmol·µL^−1^	Concentration nmol·µL^−1^ ± 4.5 nmol·µL^−1^	Concentration nmol·µL^−1^ ± 0.15 nmol·µL^−1^
Au(E)	12.6	8.9	0.35
Au(E) dex6	10.4	10.2	*
Au(E) dex40	14.2	9.5	*
Au(E) dex100	24.2	12.2	0.42
Au200	36.5	9.3	0.21
Au200 dex6	24.6	12.1	*
Au200 dex40	42.1	5.4	*
Au200 dex100	20.4	14.4	*

* Below the detection threshold of 0.15 nmol·µL^−1^.

## Supplementary Materials

The following are available online at https://www.mdpi.com/2079-4991/10/12/2499/s1, Figure S1: (a,c,d) Au(E) and (b) Au200 surfaces after Gram-staining measurements for (a,b) *L. rhamnosus* GG, (c) *L. plantarum* 299v, and (d) *L. acidophilus* cultivated 48 h, Figure S2: Results of GPC for dex6, dex40, and dex100. Experimental parameters were as follows: flow rate —1 mL·min^−1^; injection volume—100 µL; polymer concentration in the eluent—5 g·L^−1^; eluent—0.3 M Na_2_SO_4_ aqueous solution containing 0.5 M acetic acid; column—Phenomenex PolySep-SEC GFC-P Linear, Figure S3: The IR spectra for the Au(E) sample after immersion in the cationic dextran derivative, dex100, Figure S4: 2D AFM topography images of Au(E): (a) without the polymer layer and covered with (b) dex6, (c) dex40, and (d) dex100, Figure S5: Fluorescence and SEM microphotographs of *Lactobacillus plantarum* 299v after 24 h of incubation at Au(E) and Au200 substrates with and without polymer modifications, Figure S6: Fluorescence and SEM microphotographs of *Lactobacillus acidophilus* after 24 h of incubation at Au(E) and Au200 substrates with and without polymer modifications, Figure S7: Calibration curve for a lactic acid determination. Red line—linear fitting; *y* = 0.1285*x* + 0.0307 (R^2^ = 0.997).
