# Supplementary material for: Growth of Lactic Acid Bacteria on Gold—Influence of Surface Roughness and Chemical Composition
Source: Nanomaterials (Basel). 2020 Dec 13;10(12):2499. doi: 10.3390/nano10122499 (PMC7763910; doi:10.3390/nano10122499)
Supplement: Supplementary file 1 [file nanomaterials-10-02499-s001.pdf]

Supplementary Materials: Growth of Lactic Acid Bacteria on Gold—Influence of Surface Roughness and Chemical Composition

Joanna Grudzień, Magdalena Jarosz, Kamil Kamiński, Mirosława Kobasa, Karol Wolski, Marcin Kozieł, Marcin Pisarek and Grzegorz D. Sulka

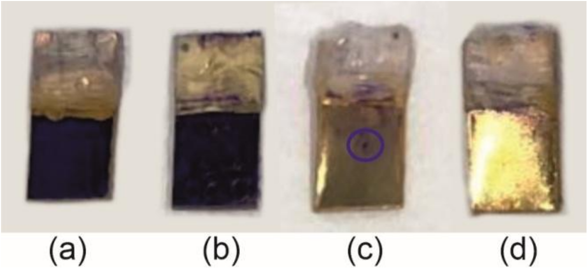

Figure S1. (**a**, **c**, **d**) Au(E) and (**b**) Au200 surfaces after Gram-staining measurements for (**a**, **b**) *L. rhamnosus* GG, (**c**) *L. plantarum* 299v, and (**d**) *L. acidophilus* cultivated 48 h.

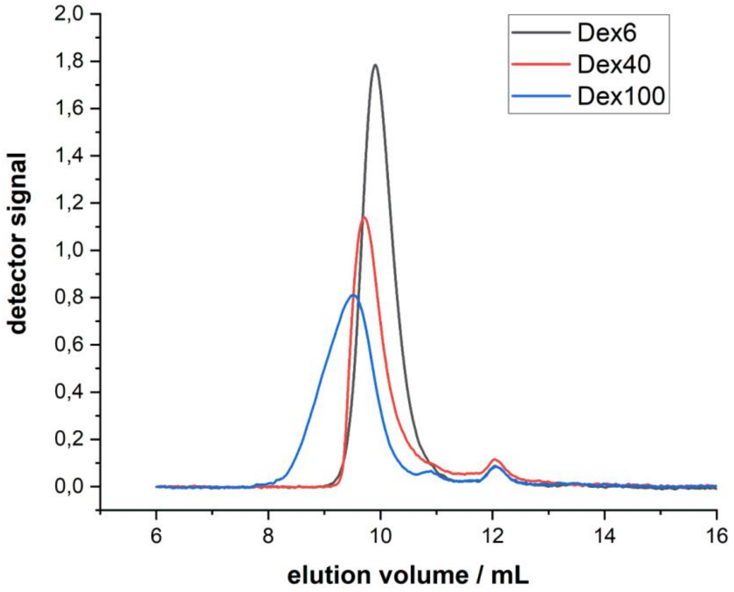

Figure S2. Results of GPC for dex6, dex40, and dex100. Experimental parameters were as follows: flow rate – 1 mL·min^{-1}; injection volume – 100 μL; polymer concentration in the eluent – 5 g·L^{-1}; eluent – 0.3 M Na_2SO_4 aqueous solution containing 0.5 M acetic acid; column – Phenomenex PolySep-SEC GFC-P Linear

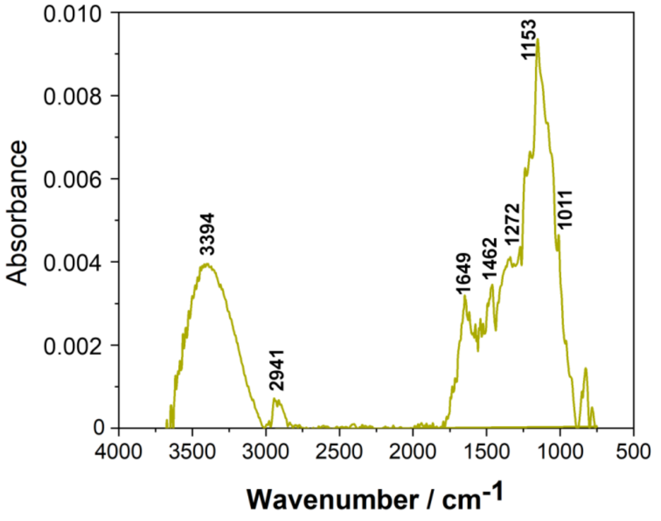

Figure S3. The IR spectra for the Au(E) sample after immersion in the cationic dextran derivative, dex100.

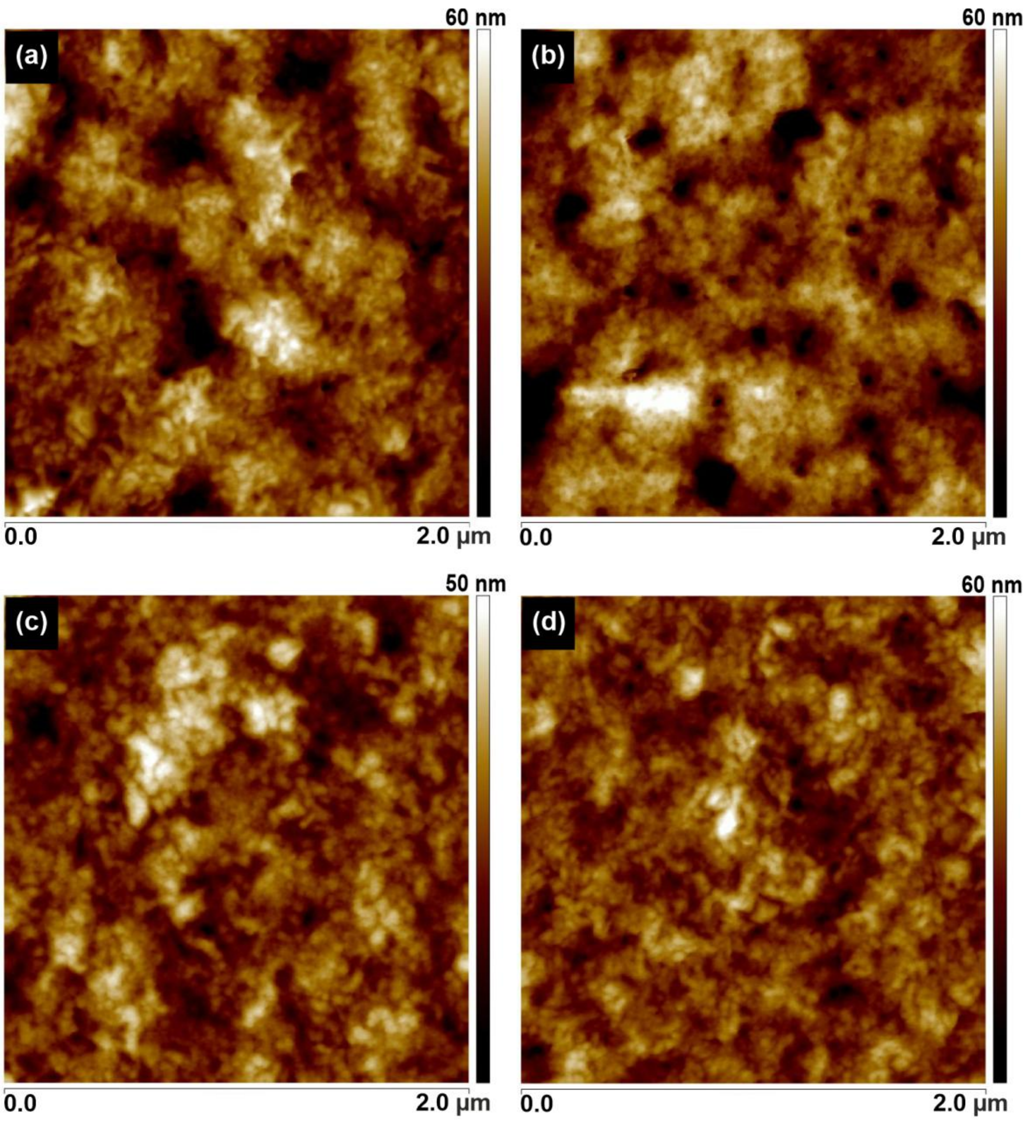

Figure S4. 2D AFM topography images of Au(E): (**a**) without the polymer layer and covered with (**b**) dex6, (**c**) dex40, and (**d**) dex100.

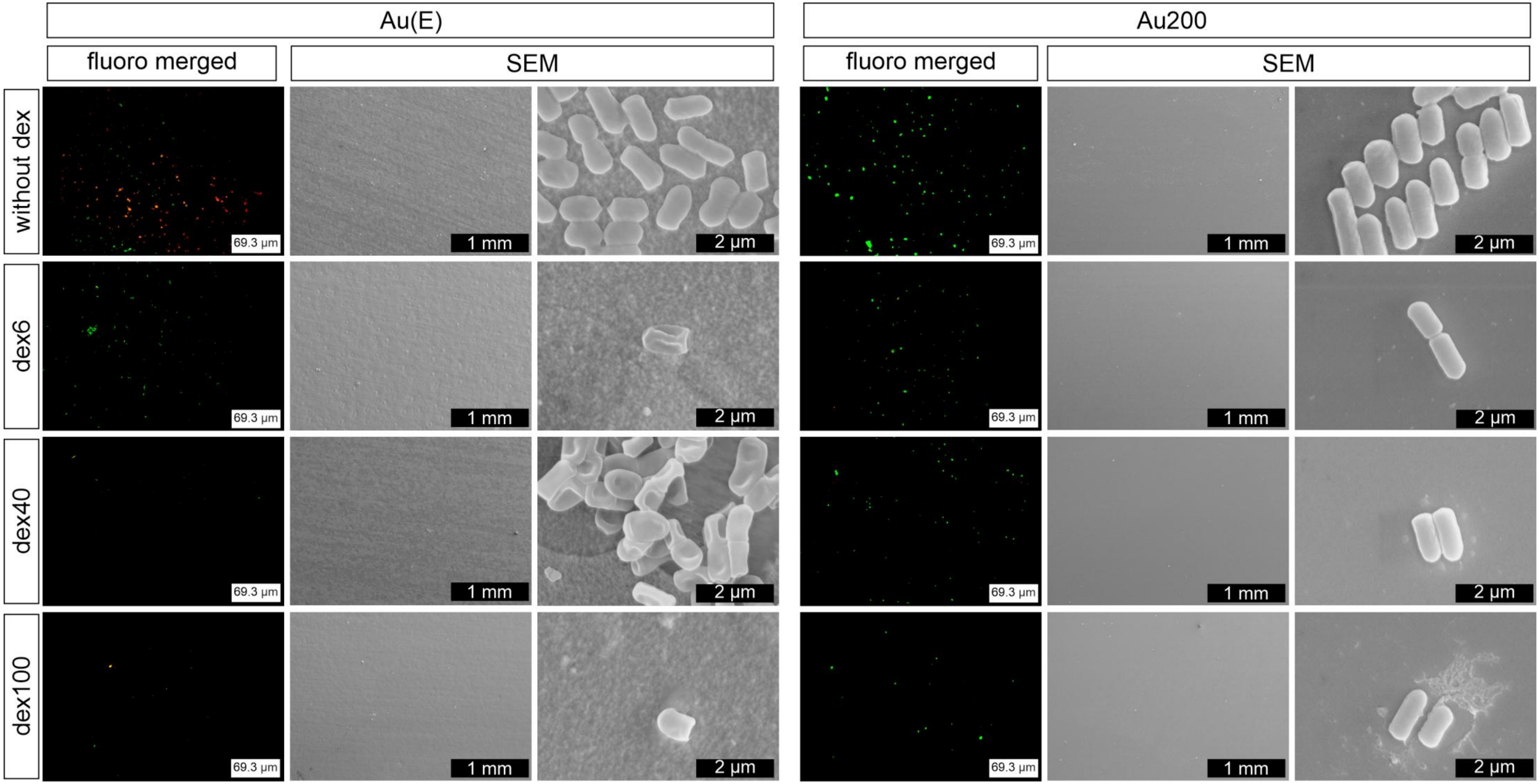

Figure S5. Fluorescence and SEM microphotographs of *Lactobacillus plantarum* 299v after 24 h of incubation at Au(E) and Au200 substrates with and without polymer modifications.

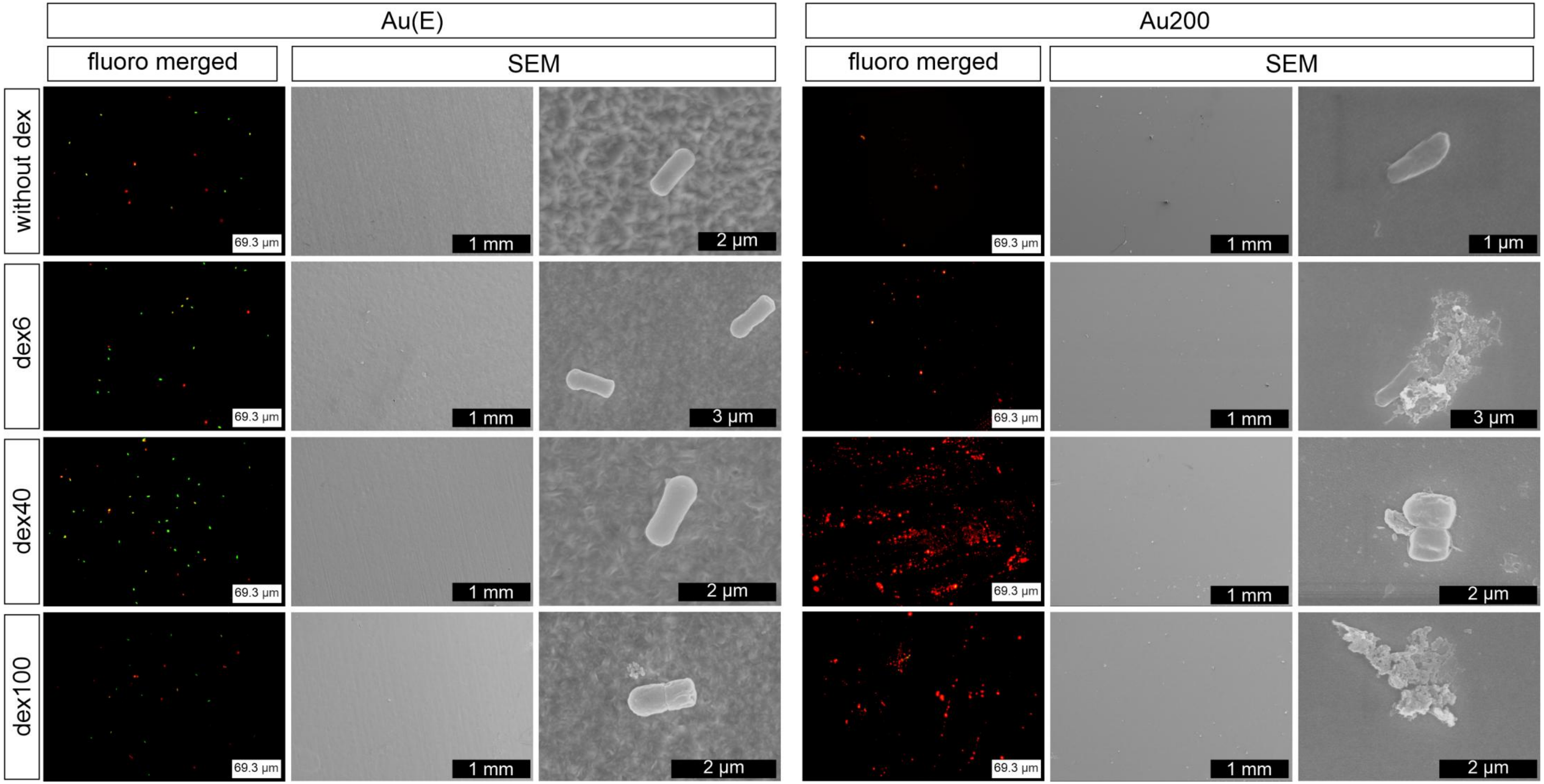

Figure S6. Fluorescence and SEM microphotographs of *Lactobacillus acidophilus* after 24 h of incubation at Au(E) and Au200 substrates with and without polymer modifications.

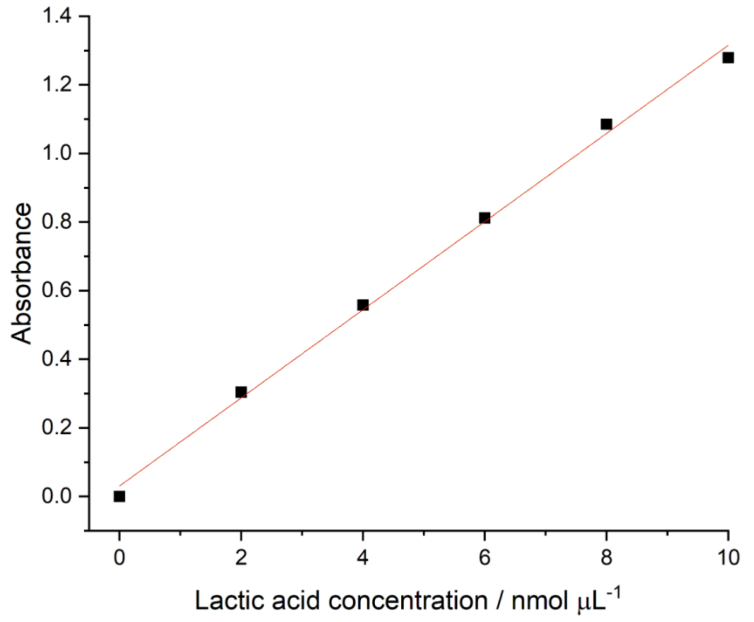

Figure S7. Calibration curve for a lactic acid determination. Red line – linear fitting; y= 0.1285x + 0.0307 (R^2 = 0.997).
